# Hepatitis E virus infection increases the risk of diabetes and severity of liver disease in patients with chronic hepatitis C virus infection

**DOI:** 10.6061/clinics/2021/e3270

**Published:** 2021-11-17

**Authors:** Patricia Momoyo Yoshimura Zitelli, Michele Gomes-Gouvêa, Daniel F. Mazo, Julio da Motta Singer, Claudia P.M.S. Oliveira, Alberto Queiroz Farias, João Renato Pinho, Ryan Yukimatsu Tanigawa, Venâncio Avancini Ferreira Alves, Flair José Carrilho, Mário Guimarães Pessoa

**Affiliations:** I Divisao de Gastroenterologia e Hepatologia Clinica, Departamento de Gastroenterologia, Hospital das Clinicas HCFMUSP, Faculdade de Medicina, Universidade de Sao Paulo, Sao Paulo, SP, BR.; II Departamento de Patologia, Faculdade de Medicina FMUSP, Universidade de Sao Paulo, Sao Paulo, SP, BR.; III Departamento de Estatistica, Instituto de Matematica e Estatistica, Universidade de Sao Paulo, Sao Paulo, SP, BR.; IV Divisao de Gastroenterologia (Gastrocentro), Faculdade de Ciencias Medicas, Universidade Estadual de Campinas (UNICAMP), Campinas, SP, BR.

**Keywords:** Hepatitis E, Hepatitis C, Chronic, Diabetes Mellitus, Liver Cirrhosis

## Abstract

**OBJECTIVES:**

Co-infection with hepatitis A or B viruses may aggravate liver injury in patients infected with hepatitis C virus (HCV). However, few studies have assessed co-infection with hepatitis E virus (HEV) and HCV. Therefore, this study aimed to assess the prevalence and impact of HEV infection among Brazilian patients with chronic HCV infection.

**METHODS:**

This observational study included adult patients with chronic HCV infection who were naive to antiviral therapy from January 2013 to March 2016. A total of 181 patients were enrolled, and HEV serology and PCR were performed for all patients.

**RESULTS:**

Seropositivity for anti-HEV IgG was detected in 22 (12.0%) patients and anti-HEV immunoglobulin M in 3 (1.6%). HEV RNA showed inconclusive results in nine (4.9%) patients and was undetectable in the remaining patients. HEV serology positive patients had more severe liver disease, characterized by liver fibrosis ≥3 *versus* ≤2 (*p*<0.001), Aspartate Aminotransferase-to-Platelet Ratio Index of ≥1.45 (*p*=0.003), and Fibrosis-4 score of ≥3.25 (*p*=0.001). Additionally, the odds of HEV-positive patients developing diabetes mellitus were 3.65 (95% CI 1.40-9.52) times the corresponding odds of HEV-negative patients. A case-control-based histological analysis (n=11 HEV-HCV-positive patients and n=22 HCV-positive patients) showed no significant differences between the groups.

**CONCLUSIONS:**

This prevalence is higher than that reported in previous studies of the general population in Brazil. Thus, HEV infection may influence the severity of liver disease and may represent an additional risk of developing diabetes mellitus in patients with HCV infection.

## INTRODUCTION

Hepatitis E virus (HEV) is characterized by a single-stranded RNA virus of variable prevalence, which belongs to the *Hepeviridae* family. Four major HEV genotypes have been reported. Genotypes 1 and 2 cause acute and epidemic diseases in humans and are more frequent in developing countries, where poor sanitary conditions prevail, either by fecal-oral transmission or from contaminated water or food. Genotypes 3 and 4 are typically transmitted via zoonosis, raw or undercooked pork consumption, or wild animal meat consumption (1-5).

The clinical course is benign with mild and self-limited symptoms in most cases, as reported by the initial series of cases among travelers to endemic regions of Asia, Africa, and the Middle East. Chronification is more likely to occur in immunocompromised hosts (6).

Previous studies have shown that patients with chronic hepatitis C virus (HCV) infection may have liver injury aggravated by co-infection with hepatitis A virus or hepatitis B virus (HBV) (7). However, few studies have evaluated the impact of HEV infection in HCV-infected patients. A study from Turkey demonstrated that among 190 HBV-infected patients (13.7%), 174 HCV-infected patients (54%), and 178 healthy individuals (15.7%) had anti-HEV IgG antibody positivity for HEV. Therefore, the screening and development of educational programs aimed at the prevention of this infection in Brazilians is justified (8). Data on the HEV role in HCV-infected patients in Brazil are scarce, whereas HEV is relatively uncommon, with prevalence of 1%-12.9%. In 1995, Pang et al. first reported data regarding HEV infection in Brazil and identified HEV seroprevalence in 6% of gold mine workers in Amazonas (9). Recent studies have shown similar blood donor results, with prevalence of 4.3% and 9.8% in Rio de Janeiro and São Paulo, respectively (10,11). More recent data have shown prevalence of 6% in the adult population, 7% in blood donors, and 3% in the general population (12).

HEV has become a public health issue, with a significant increase in morbidity and mortality worldwide. A meta-analysis study in developed countries performed on blood donors showed the seroprevalence of anti-HEV IgG antibodies using the Wantai HEV IgG ELISA kit, with 52.5%, 39.1%, 34%, and 22.4% in different regions of France, 49.6% in Poland, 49% in Italy, and 31% in the Netherlands. Three categories of risk were demonstrated: high risk (France, the Netherlands, and Poland), medium risk (Austria, Denmark, Norway, Spain, the United Kingdom, and the United States), and low risk (Canada, Scotland, Ireland, Australia, and New Zealand) (10). The main characteristics associated with high seroprevalence were advanced age; male sex; contact with animals (pig farm workers or persons with occupational exposure to pigs); raw or undercooked pork consumption; and frequent consumption of beef, smoked meats, and oysters. Low prevalence is associated with bottled water consumption (10).

Thus, we conducted this study to assess the prevalence and impact of positive markers of HEV on the severity of liver fibrosis and morbidity among Brazilian patients with chronic HCV infection.

## METHODS

### Patients and study design

This was an observational cross-sectional study of adult patients with chronic HCV infection who were naive to antiviral treatment and followed up at the outpatient liver clinic of the Department of Gastroenterology of the University of São Paulo School of Medicine. This service provides tertiary health care for a broad population of 18 million people. Patients were prospectively and consecutively recruited from January 2013 to March 2016.

We included those patients whose recent data suggested that interferon and ribavirin (standard HCV treatment at that moment) could also treat HEV infection, with HCV RNA positivity for at least 6 months, and with no previous antiviral HCV treatment (5). In contrast, we excluded those with HBV or human immunodeficiency virus co-infection, other chronic liver diseases including alcoholic liver injury and non-alcoholic fatty liver disease), use of immunosuppressive therapy, or refusal to participate in the study.

**Figure 1:**
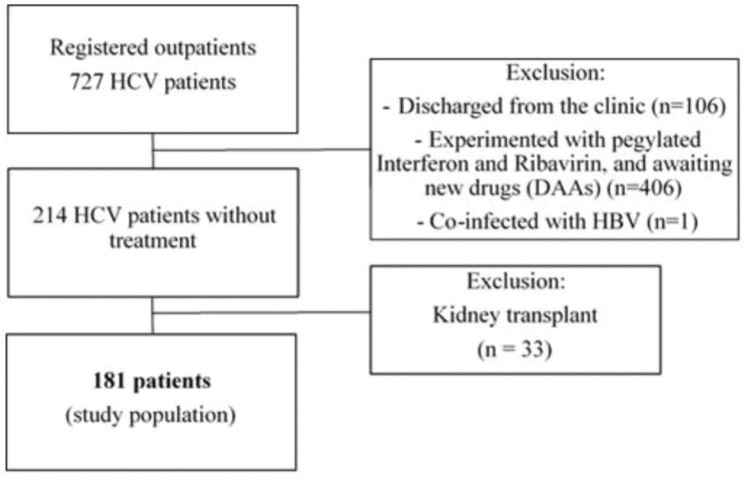
Flowchart of recruitment of patients. HCV, hepatitis C virus; DAA, direct-acting antiviral; HBV, hepatitis B virus.

We evaluated a total of 727 HCV-seropositive patients for enrollment. Among them, we excluded 106 patients discharged from the clinic, 406 who received antiviral therapy, and one with HBV co-infection. Then, we also excluded the 214 patients naive for HCV treatment and 33 who underwent kidney transplantation because they were under immunosuppressive therapy. Finally, a total of 181 patients were included for analysis (Figure 1).

Baseline demographic, clinical, and laboratory data were obtained from all participants. They included complications of liver disease (ascites, esophageal varices, and hepatic encephalopathy) and associated diseases.

### Liver fibrosis assessment

Of the 181 patients included in the study, 96 underwent liver biopsy according to routine clinical care for HCV-infected patients. We performed a case-control-based histological analysis encompassing the 11 cases of HEV-positive markers in patients who underwent a liver biopsy to analyze whether any histological characteristics were specific to HEV infection. In addition, they were matched for age, sex, HCV genotype, and severity of liver fibrosis on liver biopsy with 22 HEV-negative markers in HCV-positive patients.

Liver biopsy slides were analyzed to compare the histological aspects of HCV infection in individuals with HCV infection and positive serological markers for HEV. These cases were reviewed by an experienced liver pathologist, who was blinded to the HEV serological status and reclassified according to the METAVIR score as follows: F0, no fibrosis; F1, portal fibrosis without septa; F2, portal fibrosis and few septa extending into lobules; F3, numerous septa extending to adjacent portal tracts or terminal hepatic venules; and F4, cirrhosis. In addition, the following histological parameters were registered: portal inflammatory infiltrate, interface and parenchymal activity, steatosis, ballooning, Mallory hyaline, portal alterations, cholestatic changes, and lobular inflammatory cells.

For liver fibrosis evaluation, non-invasive methods were applied at the time of HEV serology assessment. Liver stiffness was determined using FibroScan^®^ 402 (EchoSens, Paris, France) equipped with a standard M probe. Advanced liver fibrosis (≥F3) was defined as a liver stiffness measurement of >9.5 kPa. The Aspartate Aminotransferase-to-Platelet Ratio Index (APRI) score was also computed using this formula: {[aspartate aminotransferase level/upper limit of normal] 100}/platelet count 10^9^/L. APRI values of ≥1.45 were interpreted as advanced hepatic fibrosis. Fibrosis-4 index (FIB-4) score was computed using the formula [(age]×aspartate aminotransferase level)/(platelet count 10^9^/L)×(√alanine aminotransferase level)], and FIB-4 values of ≥3.25 were interpreted as advanced hepatic fibrosis.

### HEV assessment

HEV antibodies [immunoglobulin (Ig) M and IgG] were investigated in serum samples using commercial ELISA kits (RecomWell HEV, Mikrogen GmbH, Neuried, Germany). Immunoblotting was performed using RecomLine Kits (Mikrogen GmbH) to confirm indeterminate ELISA results. The occurrence of ongoing HEV infection was investigated in all patients using quantitative real-time reverse transcription-polymerase chain reaction (qRT-PCR). HEV RNA was extracted from plasma using the QIAmp® MinElute® Virus Spin Kit (QIAGEN, Hilden, Germany). HEV RNA was eluted in 60 μL buffer, and 5 μL was used for amplification by one-step real-time PCR using the QuantiFast Pathogen RT-PCR + IC kit (QIAGEN), primers and a TaqMan probe, which targets the highly conserved ORF3 region (13). A 95% detection limit was calculated by probit analysis using 12 replicates of serial dilutions according to the WHO international standard (6329/10, Paul Ehrlich Institute, Germany) to obtain volumes of 25,000, 2,500, 250, 200, 150, 100, 50, 25, and 2.5 HEV RNA international units (IU)/mL. The detection limit predicted for this PCR was 240 IU/mL (95% confidence interval: 173-513). Patient samples were tested in triplicate with negative controls included in each run, in addition to serial dilutions of the HEV reference standard. For HEV RNA real-time PCR sample amplification with undefined results, the Eurobio® (Les Ulis, France) commercial kit was used. All assays were performed according to the manufacturer’s instructions. The staff involved in the HEV laboratory test was blinded to the patients’ clinical, biochemical, or histological data.

### Ethical considerations

The study was performed in accordance with the principles of the Declaration of Helsinki. The protocol was approved by the institutional ethics board (number 138.009), and written informed consent was obtained from all participants.

### Statistical analysis

Categorical variables are summarized as absolute and relative frequencies and continuous variables with median and interquartile ranges. Pearson chi-squared tests were performed to compare proportions, where appropriate. T-tests with Welch's corrections were performed to compare the means of continuous variables. Further, logistic regression analysis was performed to assess the impact of HEV infection on the occurrence of diabetes mellitus. Woolf test of homogeneity of strat odds ratios (case *versus* control) and Cochran-Mantel-Haenszel test were performed to compare histological variables. Statistical analyses were conducted using the R statistical software (25).

## RESULTS

Anti-HEV IgG was detected in 22 (12.0%) patients and anti-HEV IgM in 3 (1.6%). Only one patient (1.8%) was seropositive for both anti-HEV IgG and IgM antibodies (Table 1). HEV RNA quantification by qRT-PCR showed weak evidence of amplification in nine (4.9%) patients, a result considered inconclusive. These nine samples were further tested by quantitative PCR amplification using the Eurobio^®^ commercial kit, and all of them showed negative results for HEV RNA.

Study participants were divided into the mono-infected HCV group and HCV with HEV marker-positive group (HEV-HCV group). As shown in Table 2, no significant differences in baseline characteristics were detected between groups, except the higher prevalence of diabetes mellitus in the HEV-HCV group (21.0% *versus* 52%, *p*=0.003) and differences in platelet count (*p*<0.001).

A multiple logistic regression model suggested that the odds of positive HEV serology for patients with diabetes was 3.65 (95% CI 1.40-9.52) times the corresponding odds for patients without diabetes with the same severity of liver fibrosis, regardless of age. Furthermore, the odds of positive HEV markers for patients with grade F3/F4 fibrosis was multiplied by 3.91 (95% CI 1.38-11.06) for patients with diabetes, regardless of age (Table 3).

Data regarding liver disease severity as defined by the grade of liver fibrosis, clinical complications of cirrhosis, and Model For End-Stage Liver Disease (MELD) and Child-Pugh scores are summarized in Table 4. Liver fibrosis evaluation using FibroScan^®^ was performed in 122 of 181 patients, whereas APRI and FIB-4 scores were calculated for 180 and 179 patients, respectively. No significant differences between groups regarding Child-Pugh or MELD scores, prevalence of esophageal varices, or frequency of hepatic encephalopathy and ascites were observed. However, a significantly higher prevalence of advanced liver fibrosis, as assessed by non-invasive methods, was detected in the HEV-HCV group.

It is noteworthy that 63% and 67% of the patients in the HEV-positive group were found to have higher values of APRI (≥1.45) and FIB-4 (≥3.25) than 30% and 29% in the HCV mono-infected group, respectively (*p*=0.003 and *p*=0.001, respectively).

In the HEV-positive group, 18 patients (75%) had a higher fibrosis grade than 61 patients (39%) in the HCV mono-infected group (*p*=0.002), independent of the method used to classify liver fibrosis, confirming the more severe grade of liver fibrosis.

The case-control-based histological analysis (n=11 HEV-HCV and n=22 HCV patients) showed no significant differences between groups for the following parameters: portal inflammatory infiltrate, interface activity, parenchymal activity, steatosis, steatohepatitis, ballooning, Mallory hyaline, portal and lobular infiltrate (plasma cells, eosinophils, and neutrophils), biliary changes, and Kupffer cell siderosis.

## DISCUSSION

In this study of naive HCV-infected patients, we found an association between HEV infection markers (anti-HEV IgM and IgG) and comorbidities and severity of a liver disease. The 12% of patients in this study showed HEV infection, which was significantly associated with a higher frequency of diabetes mellitus and advanced liver fibrosis. However, a comprehensive liver histological analysis did not suggest any particular findings of HEV infection in these patients.

In Brazil, the prevalence of HEV infection in blood donors and the general population is 3%-7% (4). In the present study, the seroprevalence of HEV was 12% among patients with chronic HCV infection and without previous antiviral treatment. This prevalence is higher than that reported in previous studies of the general population in Brazil. Recently, Bricks et al. reported a similar HEV seroprevalence of 10.2% in patients with chronic HCV infection. However, they mention that 37.4% of patients were treated for HCV infection, which may underestimate this prevalence since pegylated interferon, ribavirin, and other antivirals can eliminate HEV (13).

HEV RNA amplification with real-time PCR showed inconclusive results for nine patients, not allowing us to ascertain the positivity of infection. Therefore, we re-evaluated the corresponding blood samples using the HEV Eurobio® commercial kit and obtained negative results. Differences in sensitivity and specificity between various commercial serological kits and HEV RNA PCR assays have been reported (14). The lack of a suitable gold standard method for diagnosing HEV infection hampers the distinction between true-positive and false-positive cases (15-17). Furthermore, HEV antibodies lack neutralizing activity, and re-infection may occur in both immunocompromised and immunocompetent hosts. Norder et al. compared five serological assays for HEV detection based on the following commercial kits: Mikrogen, DSI, Euroimmun, Axiom, and DiaPro. They noted that despite the high sensitivity for anti-HEV detection, serum samples from immunocompetent patients with HEV RNA were detected without serological markers for HEV (18).

HCV infection is associated with several conditions or extrahepatic manifestations. Among these, diabetes has been studied in depth (19). In addition, HCV infection can induce changes in insulin signaling, causing a blockage in the release and action of insulin and thereby preventing its role in regulating glucose metabolism in the body, that is, it directly promotes insulin resistance (20). In our study, an association was found between positive HEV serology and diabetes mellitus, controlled for age and severity of liver fibrosis, with an odds of 3.65 (95% CI 1.40-9.52) times the corresponding odds in those without diabetes but with the same severity of liver fibrosis, regardless of age. Furthermore, the odds of positive HEV markers for patients with grade F3/F4 fibrosis was multiplied by 3.91 (95% CI 1.38-11.06) for patients with diabetes, regardless of age.

Another Brazilian study recently reported interesting results related to glycemic status and HEV infection. The authors showed an association between insulin resistance and the presence of HEV antibodies, adjusted for age, body mass index, and cirrhosis (odds ratio=4.39; *p*=0.045) (21). This indicates that HCV infection may increase the risk of developing diabetes, but the HEV-positive markers in HCV patients appear to increase this risk further.

In this study, METAVIR grade 3 and 4 hepatic fibrosis and liver stiffness measurements and higher values of a non-invasive index to predict significant fibrosis using APRI and FIB-4 were more frequent in HEV-positive patients than in HEV-negative patients (*p*=0.002). Other studies have observed similar results, showing that patients with underlying liver disease have a worse prognosis when infected with HEV. For example, a study conducted in the UK and France tested 343 patients with chronic liver disease and showed that 3.2% (n=11) of the patients had acute hepatitis E infection with anti-IgM or HEV PCR positivity, three of whom died. In addition, the authors observed seropositive anti-IgG antibodies in 64 of 321 (19.9%) patients (22).

Wallace et al. analyzed 511 HEV-infected patients in a multicenter retrospective study in Scotland. Among them, 11% (n=58) had pre-existing cirrhosis and 21% (n=110) had diabetes. Seventeen (3.3%) HEV-related deaths were recorded, and the following factors were associated with mortality: hematological malignancy (*p*=0.005), cirrhosis (*p*=0.006), higher serum bilirubin level (*p*=0.011), and chronic HEV infection (*p*<0.001). Further, 35 (6.7%) patients developed acute hepatitis, acute-on-chronic liver failure, or acute decompensation of cirrhosis, with two patients requiring liver transplantation. The authors concluded that cirrhosis and diabetes were significantly associated with symptomatic HEV infection. The presence of underlying liver disease worsens prognosis (23).

A study conducted in China showed more complications and hepatic failure when patients with chronic HBV and HEV superinfection were than patients with chronic HBV and HAV superinfection (94.9% *versus* 61.5%, *p*<0.001, and 39.7 *versus* 11.5%, *p*=0.002, respectively). Furthermore, they demonstrated higher mortality in the chronic HBV plus HEV group (33.8 *versus* 1.9%, *p*<0.001) (24).

A limitation of the present study is that we could not determine the time of HEV or HCV infection. These infections are usually asymptomatic, and patients with HEV infection are only viremic for 2 weeks. Furthermore, the discrepancy of differences in sensitivity and specificity between the various commercial serological kits and HEV RNA PCR assays makes it difficult to diagnose HEV infection.

In summary, the seroprevalence of HEV was high in this study, and HEV infection may influence the severity of liver disease and represent an additional risk of developing diabetes mellitus and liver decompensation in patients with HCV infection.

## AUTHOR CONTRIBUTIONS

Pessoa MG conceived and designed the study, cared for the patients, contributed to the data analysis and interpretation, and wrote and reviewed the manuscript. Zitelli PMY collected and assembled the data, processed the biological samples, contributed to the analysis and interpretation, and wrote the manuscript. Gomes-Gouvêa M and Pinho JR processed the HEV laboratory test and analyzed the results. Singer JM contributed to the statistical analysis and interpretation of the results. Mazo DF, Oliveira CPMS, Farias AQ, and Carrilho FJ cared for the patients and critically reviewed the manuscript. Tanigawa RY and Alves VAF histologically assessed liver biopsy slides. All authors critically revised the manuscript, approved the final version to be published, and agreed to be accountable for all aspects of the work.

## Figures and Tables

**Table 1:** Frequency of patients with chronic hepatitis C with different combinations of HEV test results.

Real-time	Anti-HEV IgM	Anti-HEV IgG	Frequency
Negative	Negative	Negative	157
Negative	Negative	Positive	21
Negative	Positive	Negative	2
Negative	Positive	Positive	1
Total			181

HEV, hepatitis E virus; Ig, immunoglobulin.

**Table 2:** Demographic, clinical and laboratorial exams characteristics of patients with anti-HEV-positive and -negative antibodies.

	HCV group		HEV-HCV group		
Variable	n/total	mean±SD or %	n/total	mean±SD or %	*p*-value
Age (years)	157	52.5±12.9	24	57.0±10.4	0.070
Male	73/157	47%	9/24	38%	0.546
Weight (kg)	150	71.8±15.3	23	73.4±23.3	0.757
Height (cm)	148	164.5±9.9	23	161.2±12.7	0.243
BMI (kg/m^2^)	148	28.5±5.1	23	27.9±6.8	0.343
Arterial hypertension	68/151	45%	13/23	57%	0.421
Diabetes mellitus	32/151	21%	12/23	52%	0.003*
Dyslipidemia	22/151	15%	3/23	13%	1
Glucose	148	102.9±49.4	23	128.7±78.7	0.140
Insulin	48	15.4±10.6	6	26.4±18.4	0.208
HOMA	46	4.2±3.4	6	11.0±10.1	0.158
Albumin (g/dL)	154	4.2±0.5	24	4.0±0.6	0.096
Alpha-fetoprotein (ng/mL)	147	53.5±323.0	23	14.2±29.8	0.153
ALT (U/L)	157	65.6±48.1	24	61.8±52.1	0.739
AST (U/L)	157	60.5±49.2	24	67.3±48.5	0.527
Total bilirubin (mg/dL)	157	0.8±0.6	24	0.9±0.4	0.171
Creatinine (mg/dL)	155	1.3±1.8	24	1.2±2.0	0.864
Platelets (10^3^/mm^3^)	156	182.1±75.6	24	116.5±59.3	<0.001*
Hemoglobin (g/dL)	156	14.0±1.8	24	13.2±2.5	0.111
INR	153	1.1±0.3	24	1.1±0.2	0.723

ALT, alanine aminotransferase; AST, aspartate aminotransferase; BMI, body mass index; HCV, hepatitis C virus; HEV, hepatitis E virus; HOMA, homeostasis model assessment; INR, international normalized ratio; SD, standard deviation; *p<0.05.

**Table 3:** Results of multiple logistic regression model with HEV positivity as response variable and diabetes mellitus, age, and grade of liver fibrosis (F0/F1/F2 or F3/F4) as explanatory variables.

Coefficient	Estimate	Standard error	*p*-value
Intercept	-3.11	1.16	0.01*
Diabetes mellitus	1.30	0.49	0.01*
Fibrosis F3/F4	1.36	0.53	0.01*
Age	-0.0002	0.02	0.02*

HEV, hepatitis E virus. *p<0.05.

**Table 4:** Comparison of HEV-HCV patients and HCV mono-infected patients regarding liver disease severity.

	HCV group		HEV-HCV group		
Variable	n/total	mean±SD or %	n/total	mean±SD or %	*p*-value
Grade of fibrosis (F3/F4)	61/157	39%	18/24	75.0%	0.002*
FibroScan >9.5 kPa	39/110	36%	8/12	67%	0.072
APRI ≥1.45	46/156	30%	15/24	63%	0.003*
FIB-4 score ≥3.25	44/154	29%	16/24	67%	0.001*
Ascites	12/44	27%	6/15	40%	0.549
Encephalopathy	5/44	11%	3/15	20%	0.684
Esophageal varices	33/44	75%	11/15	73%	1
Child-Pugh A	31/44	71%	9/15	60%	0.668
MELD	43	10.3±3.5	15	9.2±1.6	0.109

APRI, AST to platelet ratio index; FIB-4, Fibrosis-4; HCV, hepatitis C virus; HEV, hepatitis E virus; SD, standard deviation; MELD, Model For End-Stage Liver Disease. *p<0.05.
